# Dissociative Bipolar At-Risk Phenotype: Traumatic Burden and Subthreshold Affective Psychopathology in a Help-Seeking Youth Sample

**DOI:** 10.3390/brainsci16040349

**Published:** 2026-03-25

**Authors:** Francesca Scopetta, Marta Barbi, Gianmarco Cinesi, Filippo De Giorgi, Alfonso Tortorella, Giulia Menculini

**Affiliations:** 1 Section of Psychiatry, Department of Medicine and Surgery, University of Perugia, 06132 Perugia, Italy; francesca.scopetta@yahoo.it (F.S.); martabarbi21@libero.it (M.B.); gianmarco.cinesi@gmail.com (G.C.); alfonso.tortorella@unipg.it (A.T.); 2 Division of Psychiatry, Clinical Psychology and Rehabilitation, General Hospital of Perugia, 06132 Perugia, Italy; filippo.degiorgi@ospedale.perugia.it

**Keywords:** dissociation, bipolar at-risk, youth, trauma, cyclothymic temperament, early intervention

## Abstract

**Highlights:**

**What are the main findings?**

- Bipolar at-risk (BAR) status and clinically significant dissociation were highly prevalent and frequently co-occurred in this clinically enriched, second-level outpatient sample of help-seeking youths. Within the BAR subgroup, youths with dissociative symptoms reported greater traumatic burden, more severe depressive symptoms, and higher anxious temperament scores.
- Attentional impulsivity did not mediate the relationship between dissociation and affective vulnerability, suggesting a direct link between dissociative symptoms and bipolar-spectrum risk.

**What is the implication of the main finding?**

- Integrating systematic assessment of dissociation into early-intervention pathways may improve risk stratification for adolescents and young adults presenting with affective instability.

**Abstract:**

Background: Youth mental health services increasingly encounter adolescents and young adults with complex affective presentations and trauma histories. Dissociation has been proposed as a clinically relevant marker within bipolar vulnerability pathways but remains underrecognized in early-intervention settings. This pilot study investigated the prevalence and clinical correlates of bipolar at-risk (BAR) status in a help-seeking youth sample, with specific focus on dissociative symptoms in this vulnerable population. Methods: A pilot study with a cross-sectional design was conducted in a specialized outpatient clinic for 14–25-year-olds. Seventy-six participants without Diagnostic and Statistical Manual of Mental Disorders, Fifth Edition, Text Revision bipolar disorder completed a multidimensional assessment, including dissociative (Dissociative Experiences Scale version 2 [DES-II], Adolescent-DES [A-DES], Structured Clinical Interview for DSM Dissociative Disorders [SCID-D]), affective, anxiety, impulsivity, and prodromal symptom measures. BAR status (BAR+) was determined via clinical interview according to Bechdolf criteria. Clinically significant dissociation (DES+) was defined by established cut-offs at the DES-II and A-DES scales. Group comparisons, binary logistic regression and exploratory mediation analysis were performed. Results: In our sample, 44.7% of the participants met BAR+ criteria and 42.9% displayed clinically significant dissociation. Patients with BAR+ status more frequently reported familiar history of affective disorders, previous antidepressant use, loneliness, and non-suicidal self-injury. They displayed more severe depressive symptoms and impulsivity, as well as higher scores at all the affective temperament subscale except for hyperthymic. BAR+ patients displayed higher prevalence of dissociative symptoms than BAR− (51.6% vs. 24.2%; *p* = 0.045). Among the BAR+ subgroup, DES+ youths showed greater traumatic burden, depressive symptoms, and anxious temperament scores. Dissociation was associated with BAR+ status (OR 3.2) after adjusting for age, gender, and loneliness, while attentional impulsivity did not mediate this relationship. Conclusions: Dissociative symptomatology is highly prevalent among help-seeking youths and is directly associated with subthreshold bipolar-spectrum vulnerability. A dissociative BAR phenotype, marked by emotional instability and trauma exposure, may delineate a clinically complex subgroup, supporting the integration of dissociation-focused assessment into youth bipolar-risk staging and early-intervention protocols.

## 1. Introduction

Youth mental health has become a major public health priority due to a progressive decline in psychological well-being observed among younger generations. Previous research documented a sustained increase in internalizing disorders and emotional distress since the mid-1990s, independent of the Coronavirus disease 2019 (COVID-19) pandemic [1,2]. Youth, defined by the World Health Organization (WHO) as the age range 15–24 encompassing adolescence and young adulthood, represents a critical neurodevelopmental period characterized by substantial remodeling of fronto-limbic and social-affective circuitry, which is associated with increased vulnerability to the onset of psychopathological conditions [3]. Contemporary cohorts, particularly Generation Z, show higher prevalence rates of anxiety and depressive disorders compared with previous generations, together with higher levels of multimorbidity and functional impairment [4,5]. Bipolar disorder (BD) commonly emerges during this developmental period [6]. However, early identification remains challenging. The average duration of untreated illness (DUI) in BD ranges from five to ten years [7,8], and delayed diagnosis has been associated with increased risk of suicidality, reduced treatment responsiveness, and long-term functional decline [9]. These observations have contributed to a shift from categorical diagnostic approaches toward clinical staging frameworks that emphasize the identification of subthreshold and prodromal affective features [10]. The Bipolar at-risk (BAR) criteria proposed by Bechdolf et al. [11] provide an operational approach for identifying affective vulnerability in help-seeking youths. The criteria focus on subthreshold manic symptoms, cyclothymic and depressive instability, and familial risk for BD. These domains describe individuals who present with clinically relevant affective features prior to the onset of a clear-cut, full-syndromal presentation. Prospective validation studies have supported the predictive validity of the BAR model [11]. Large-scale European research, including the Early-BipoLife project, has reported that extended BAR criteria (BARS) identify a subgroup of help-seeking youths characterized by mood instability, subsyndromal depressive symptoms, and functional impairment [12,13]. Among environmental and developmental factors associated with bipolar vulnerability, adverse childhood experiences (ACEs) have been linked to earlier illness onset and greater clinical severity [14]. In clinical populations with BD, ACEs—particularly emotional abuse and neglect—have been associated with earlier age at onset, increased affective lability, higher prevalence of mixed features, elevated suicidality, and reduced response to mood-stabilizing treatments [15,16,17]. In addition, the use of antidepressant treatments in individuals with unrecognized bipolar vulnerability has been associated with an increased risk of mood switching and mixed features [18,19]. Dissociation has been described as a relevant clinical and psychobiological construct within this context. It is defined as a disruption in the normal integration of consciousness, memory, identity, and perception [20]. Dissociative responses have been reported in association with exposure to overwhelming affective or traumatic experiences [21,22]. Empirical studies indicate that dissociative symptoms are associated with both depressive and psychotic symptomatology and may mediate the relationship between childhood adversity and later clinical outcomes [23,24,25]. Dissociation has also been linked to emotional dysregulation and avoidance-based coping strategies [26,27]. Depressive presentations with prominent dissociative features have been associated with poorer treatment outcomes [28]. Despite their potential clinical relevance, dissociative phenomena remain frequently underrecognized in clinical practice, with reported diagnostic delays ranging from 5 to 12 years [29]. Given these premises, the present study investigates the prevalence and clinical correlates of subthreshold affective vulnerability, evaluated as BAR status, in a help-seeking sample of adolescents and young adults. It further examines the interaction between BAR status and clinically significant dissociation to evaluate whether dissociative symptomatology is associated with specific phenotypes within the bipolar-risk spectrum. The expected relevance of the study results is in informing early assessment strategies and contributing to the development of prevention-oriented approaches in youth mental health services.

## 2. Materials and Methods

### 2.1. Study Design and Setting

This pilot study employed a cross-sectional design and was conducted at the Specialized Outpatient Clinic for the Assessment of Psychopathological Manifestations in Adolescence and Young Adulthood at the Division of Psychiatry of the University Hospital of Perugia, Italy. The clinic serves as a second-level diagnostic service, providing short- to medium-term assessments for adolescents and young adults presenting with complex or multidimensional psychopathological profiles. Referrals are received from Community Mental Health Centers (CMHCs), Addiction Services (Ser.D), Child and Adolescent Neuropsychiatry Services, other hospital units, as well as general practitioners, family pediatricians, and private specialists. In line with WHO conceptualizations of developmental transitions between adolescence and young adulthood, the term youth is used in our service to refer to individuals aged 14–25 years, capturing the developmental window characterized by heightened affective instability, identity formation, and emerging severe mental disorders. We slightly extended the age range compared to the WHO definition in line with current clinical practice in our services.

### 2.2. Study Procedures

As part of routine outpatient care, participants completed a structured psychodiagnostics assessment protocol conducted by psychiatrists and supervised psychiatry residents over multiple clinical sessions, typically a minimum of three, with additional sessions scheduled as needed to complete the evaluation. During the initial session, a comprehensive clinical and developmental history was obtained, and an initial set of screening instruments was administered to assess personality traits and core psychopathological domains. Subsequent sessions included an extended battery of standardized instruments designed to achieve a multidimensional and integrative clinical characterization. The standard assessment covered a wide range of symptom domains, including affective, obsessive–compulsive, psychotic-spectrum (prodromal and subthreshold), post-traumatic, and dissociative dimensions, as well as cognitive functioning and neurodevelopmental features. For the purposes of the present study, only measures that were completed by >95% of the eligible patients were included in the analysis. All collected data were entered into a dedicated database that encompassed sociodemographic variables, clinical history, and assessment test scores. The study was conducted according to good clinical practice procedures and in accordance with the Declaration of Helsinki principles. All patients or, where applicable, their parents or legal representatives, signed informed consent for participation in the study. The study was approved by the Ethical Committee of the Umbria Region (protocol n. CE-2230/25, approved on 19 February 2025).

### 2.3. Study Population

The study sample comprised adolescents and young adults aged 14–25 years, consistent with the target population of the outpatient clinic. Exclusion criteria included a documented diagnosis of intellectual disability, as defined by the Diagnostic and Statistical Manual of Mental Disorders, Fifth Edition, Text Revision (DSM-5-TR) [20], and insufficient proficiency in the Italian language, which could compromise the validity of the psychodiagnostic assessment. Participants who reached the age of 26 years during the evaluation period were not excluded if they met the inclusion criteria at initial access. Only participants who had completed and validly returned standardized instruments assessing dissociative symptomatology were included in the analysis. The screening tools used for the evaluation of dissociative symptoms were the Dissociative Experiences Scale–II (DES-II) [30] for adults and the Adolescent Dissociative Experiences Scale (A-DES) [31] for younger participants. Individuals scoring above the clinical cut-off of 30 at the DES-II [32] or 4 at the A-DES [33] were grouped as having clinically significant dissociation.

### 2.4. Assessment Instruments

A combination of self-report questionnaires and clinician-administered instruments was used to obtain a comprehensive, multidimensional assessment of psychopathological features. The evaluation protocol investigated affective symptoms (bipolar-spectrum vulnerability, anxiety and depressive symptoms, affective temperament), dissociative experiences, impulsivity, psychosis-risk features, circadian preferences, obsessive–compulsive symptoms, and neurodevelopmental traits. To note, some of the scales evaluating specific dimensions (circadian preference, obsessive-compulsive, and neurodevelopmental features) were not completed by >5% on the included patients and we thus did not include them in the analysis. Affective symptoms were assessed through both self-report and clinician-rated measures, including the Beck Depression Inventory–II (BDI-II) [34], Hamilton Rating Scale for Depression (HAM-D) [35], Montgomery–Åsberg Depression Rating Scale (MADRS) [36], and the Koukopoulos Mixed Depression Rating Scale (KMDRS) [37] for the evaluation of depressive features, the State–Trait Anxiety Inventory–Y (STAI-Y) [38] and Hamilton Anxiety Rating Scale (HAM-A) [39] for anxiety, and instruments for assessing bipolar spectrum. In particular, the presence of a clear-cut BD, which represented an exclusion criteria, was screened using the Mood Disorder Questionnaire (MDQ) [40] and the Hypomania Checklist–32 (HCL-32) [41] and subsequently confirmed with DSM-5-TR criteria. Affective temperaments were assessed with the brief TEMPS-M, Münster version [42]. Impulsivity was measured using the Barratt Impulsiveness Scale–11 (BIS-11) [43]. Psychosis-risk features were assessed using the Prodromal Questionnaire, Brief Version (PQ-B) [44]. This integrated approach, combining self-report and clinician-administered tools, ensured both subjective insight and objective clinical validation across all assessment domains.

### 2.5. Measures and Operational Definitions

In our help-seeking youth sample, BAR status was determined by two senior psychiatrists through clinical assessment according to Bechdolf criteria, based on the presence of subthreshold manic symptoms, cyclothymic features, and/or familial risk for BD. Participants meeting any BAR criterion were classified as BAR+, whereas those not fulfilling criteria were classified as BAR−.

As already underlined, clinically significant dissociative symptomatology was assessed using age-appropriate self-report instruments. Participants aged ≥ 18 years completed the DES-II, whereas adolescents completed the A-DES. Clinically significant dissociation was operationalized categorically (DES+) using established and pragmatic thresholds: a DES-II score ≥ 30 in adults and an A-DES score ≥ 4 in adolescents, reflecting elevated dissociative symptomatology across age groups. Participants scoring below these thresholds were classified as DES−. This categorical approach was adopted to harmonize dissociation status across developmentally specific instruments. Patients who were grouped as DES+ subsequently underwent the Structured Clinical Interview for DSM—Dissociative Disorders (SCID-D) [45,46].

As for the other relevant psychopathological features, we used validated psychometric assessment scales, as already defined above. Continuous scores were used for correlation and regression analyses.

Social and clinical covariates were extracted from clinical records and structured assessment by two senior psychiatrists during clinical interviews. Sociodemographic variables included age, gender, and referral pathway. Clinical variables were assessed as categorical (present/absent) by using operational definitions. As for traumatic burden, we focused on lifetime exposure to adverse experiences potentially associated with emotional dysregulation or dissociation, further characterized according to developmental timing—with specific attention to childhood, coded as up to 12 years old—and phenomenological features, e.g., interpersonal vs. non-interpersonal events. Loneliness was coded as present when participants described persistent subjective feelings of emotional isolation, lack of belonging, or perceived absence of supportive relationships, irrespective of objective social network size. Social isolation was defined as stable pattern of limited peer relationships, withdrawal from social activities, or marked reduction in interpersonal engagement compared to age-expected functioning. Digital hyperconnection was considered as present when participants or their relatives reported pervasive and time-consuming use of digital devices or online activities associated with functional impairment, sleep disruption, or reduced offline social engagement.

### 2.6. Statistical Analysis

All data were entered into a dedicated database and analyzed using IBM SPSS Statistics, version 26.0 (IBM Corp., Armonk, NY, USA). Descriptive statistics were computed to summarize the distribution of study variables. Categorical variables were expressed as absolute frequencies and percentages, and continuous variables as means and standard deviations. Sample size minimally varied across analyses due to missing data (<5%) in self-report measures; analyses were conducted using available-case data without imputation. Bivariate analyses compared (1) BAR+ vs. BAR− participants (BAR+ vs. BAR−), (2) DES+ vs. DES− participants within the BAR+ subgroup (BAR+/DES+ vs. BAR+/DES−). Between-group differences were tested using the chi-square test or Fisher’s exact test for categorical variables, and Student’s *t*-test or Mann–Whitney U test for continuous variables, depending on data distribution. Normality was verified using the Kolmogorov–Smirnov test. All analyses were two-tailed, and statistical significance was set at *p* < 0.05. We performed a binary logistic regression model to assess the association between dissociative symptomatology and BAR status. The model was controlled for sociodemographic measures, particularly age, gender, and loneliness. The latter was entered into the model since it represented an indirect measure of social functioning, presenting statistical significance in bivariate analyses. Odds ratios (Ors) and 95% confidence intervals (CI) were calculated for all the variables. Multicollinearity was assessed using tolerance (>0.1) and variance inflation factors (VIF < 10). An exploratory simple mediation model was tested using Hayes’ PROCESS Macro (Model 4) to assess whether the association between dissociative symptoms was mediated by impulsivity, specifying the BIS-11 attentional subscale as the mediator, clinically significant dissociation as the independent variable, and BAR status as the dependent variable. We included impulsivity as a mediator since it represents a measurable indicator of emotional dysregulation during youth, possibly linked to both dissociative phenomena and affective vulnerability. Given the limited sample size, multivariable and mediation analyses were considered exploratory and interpreted cautiously as hypothesis-generating.

## 3. Results

### 3.1. Sample Description

**Figure 1:**
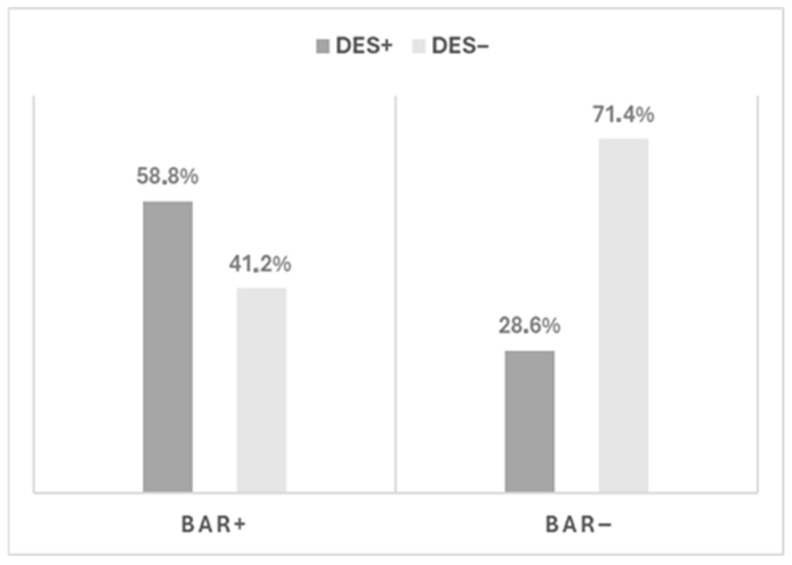
Distribution of Bipolar-at-risk (BAR) status according to dissociative symptomatology. Participants were stratified into clinically significant dissociation (DES+) and non-clinically significant dissociation (DES−) groups based on age-appropriate dissociation measures. Bars represent the proportion of individuals meeting BAR criteria (Bechdolf) within each dissociation group.

Descriptive analyses were performed on a sample of 77 patients referred to the second-level outpatient service. The sample was drawn from 262 consecutive patients, as the individuals included in the analysis had validly completed either the DES-II or the A-DES. Most participants (n = 45, 58.4%) were females, Italian (n = 76, 98.7%), with a mean age at assessment of 18.6 ± 3.1 years (median = 17). In the sample, 51.9% (n = 40) subjects were minors (<18 years). Referrals originated primarily from CMHCs (31.2%; n = 24) and general practitioners (26.0%; n = 20). Regarding social functioning, 64.9% (n = 50) of participants reported maintaining stable interpersonal relationships, whereas 33.8% (n = 26) described social isolation or active avoidance of peer interactions. Digital hyperconnection was reported by 9% (n = 7) of youths in the sample. Based on DES-II and A-DES scores, participants with clinically significant dissociation (DES+) were 42.9% of the overall sample (n = 33). Among those who completed the SCID-D, 22.2% met DSM-5-TR diagnostic criteria for a dissociative disorder. DES+ participants had lower mean age compared to DES individuals (17.84 ± 3.02 vs. 19.07 ± 3.09 years; Mann–Whitney U = 517.00, *p* = 0.088) and showed a female predominance (66.7%; n = 22; *p* = 0.402), with neither difference reaching statistical significance. Among DES+ participants, more than half met criteria for BAR status, showing a significantly higher prevalence than DES individuals (58.8% vs. 28.6%; *p* = 0.008; Figure 1).

Mean scores on the cyclothymic temperament subscale of the briefTEMPS-M were also significantly higher in DES+ participants (27.08 ± 4.79) compared to DES− individuals (18.23 ± 6.85; *p* < 0.001).

No statistically significant differences were observed between groups on the HCL-32 or MDQ scores.

### 3.2. Clinical Correlates of BAR Status

#### 3.2.1. BAR+ vs. BAR−

Following exclusion of one participant with a DSM-5-TR diagnosis of BD, analyses were conducted on a final sample of 76 individuals. BAR status was identified in 34 participants (44.7%, BAR+), with the remaining participants classified as BAR− (55.3%). Comparisons between BAR+ and BAR− participants indicated that BAR+ individuals more frequently reported a family history of affective disorders (65.6% vs. 36.8%; *p* = 0.031), particularly concerning parents (68.8% vs. 37.5%; *p* = 0.016). They also showed higher rates of lifetime anxiety disorders (79.4% vs. 54.8%; *p* = 0.045) and current or lifetime non-suicidal self-injury (NSSI) (73.5% vs. 31.0%; *p* < 0.001).

**Figure 2:**
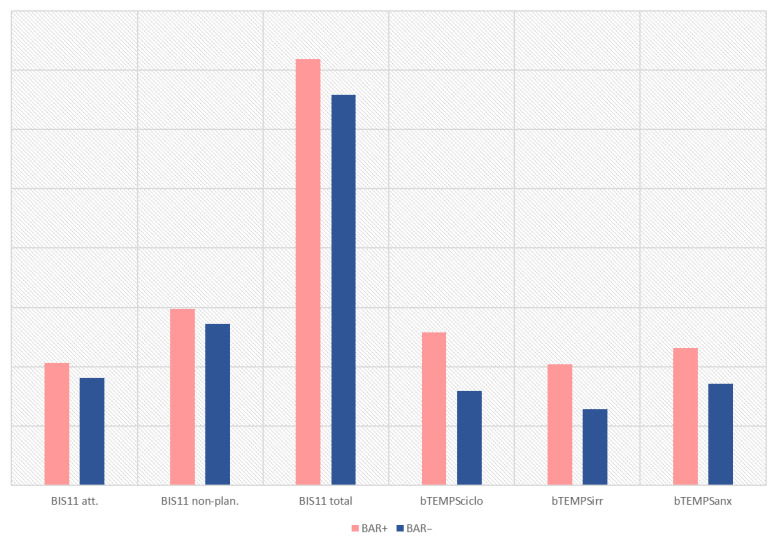
Comparison of psychopathological variables between Bipolar at-risk (BAR+) and non–at-risk (BAR−) participants. The figure displays mean scores (±SD) for impulsivity (BIS-11 subscales) and affective temperament dimensions (briefTEMPS-M) across the two subgroups.

BAR+ participants had received antidepressant treatment more frequently than BAR− individuals (29.4% vs. 9.5%; *p* = 0.037). Loneliness was also more common among BAR+ participants (48.5% vs. 21.4%; *p* = 0.026). As for psychopathological characteristics, depressive symptoms were more severe in the BAR+ population (MADRS total score 17.60 ± 8.90 vs. 11.13 ± 7.58; *p* = 0.005). Moreover, BAR+ participants showed significantly higher mean scores on the BIS-11 than BAR− individuals across multiple subscales: attentional impulsivity (20.67 ± 3.56 vs. 18.16 ± 4.73; *p* = 0.014), non-planning impulsivity (29.70 ± 5.38 vs. 27.22 ± 3.92; *p* = 0.030), and total score (71.82 ± 10.14 vs. 65.84 ± 12.36; *p* = 0.031). Regarding affective temperament, BAR+ participants had significantly higher scores on the cyclothymic (25.75 ± 5.72 vs. 15.87 ± 5.62; *p* < 0.001), irritable (20.37 ± 7.21 vs. 12.80 ± 4.99; *p* = 0.001), anxious (23.11 ± 5.91 vs. 17.13 ± 5.62; *p* = 0.004), and depressive (median 27 vs. 20; *p* = 0.038) subscales of the Brief TEMPS-M (Figure 2). Prodromal symptoms also showed a higher severity in the BAR+ subgroup (median 10 vs. 4.5; *p* = 0.015). Complete bivariate comparisons can be observed in Table 1.

#### 3.2.2. DES+ Patients Among BAR+

Within the BAR+ subgroup, clinically significant dissociation (DES+) was significantly more prevalent when compared to BAR− (51.6% vs. 24.2%; *p* = 0.045). When comparing BAR+ patients with dissociative symptoms (BAR+/DES+) to those who did not display this clinical feature (BAR+/DES−), they reported a higher frequency of type I or II traumatic events, primarily related to early-life trauma (76.5% vs. 41.2%; *p* = 0.040) and a higher prevalence of loneliness (84.6% vs. 15.4%; *p* = 0.032). Youths in the BAR+/DES+ subgroup also reported more frequent lifetime antidepressant prescription (100% vs. 0%; *p* = 0.020). As for psychopathological characteristics, mean scores at the BDI-II were 30.60 ± 12.72 for BAR+/DES+ and 16.67 ± 12.33 for BAR+/DES− participants (*p* = 0.015). They also showed significantly higher mean scores on the anxious temperament subscale of the brief TEMPS-M (26.40 ± 4.35 versus 19.44 ± 5.34, *p* = 006). Differences in mean ranks consistently indicated higher scores among BAR+/DES+ participants, with moderate effect sizes for depressive symptoms and large effects for anxious temperament. Comparisons of psychopathological characteristics as measured by the main assessment scales are reported in Table 2.

#### 3.2.3. Binary Logistic Regression

To verify the association between dissociation and BAR status, an exploratory binary logistic regression analysis was performed with BAR+ status as the dependent variable and DES+ as the main predictor, adjusting for age, gender, and loneliness. The model was statistically significant (χ^2^ (4) = 14.023; *p* = 0.007) and explained a moderate proportion of variance (McFadden R^2^ = 0.15; Cox–Snell R^2^ = 0.17; Nagelkerke R^2^ = 0.23). The logistic regression confirmed results already underlined at the mediation analysis. The effect of DES+ was significant (Wald = 4.677; *p* = 0.031), with β = 1.27 (95% CI [0.32–2.23]) and OR = 3.165, indicating that DES+ participants were approximately 3.2 times more likely to be classified as BAR+ than DES− individuals after adjusting for covariates (see Table 3). Estimated marginal probabilities confirmed this association: DES−: 0.32 (95% CI [0.20–0.47]) and DES+: 0.62 (95% CI [0.45–0.77]), representing a difference of approximately 31 percentage points (*p* = 0.006). The model showed good fit (log-likelihood = −83.04) and satisfactory discriminative ability, consistent with the mediation analysis. The use of 5000 bias-corrected bootstrap resamples improved precision and reduced Type I error risk. The sample size was considered adequate for the exploratory binary logistic regression given the effect size (OR > 3) and balanced group distribution, although it may have limited power to detect small indirect effects.

#### 3.2.4. Mediation Analysis

**Figure 3:**
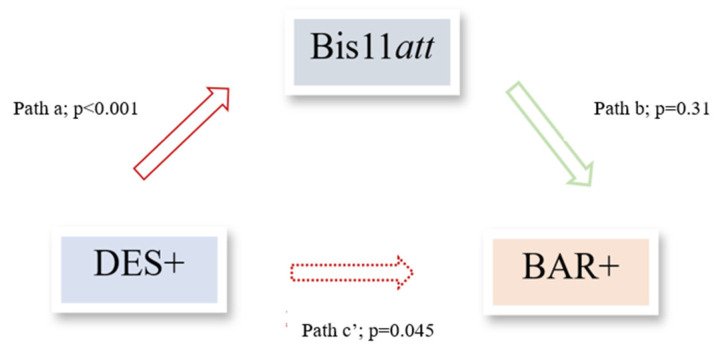
Mediation model examining the role of attentional impulsivity in the relationship between dissociative symptomatology and bipolar vulnerability. The diagram illustrates the direct effect of dissociation (DES+) on bipolar at-risk (BAR+) status and the indirect pathway via attentional impulsivity.

To determine whether attentional impulsivity mediated the relationship between dissociative symptomatology (DES+) and affective vulnerability (BAR+), an exploratory simple mediation model was estimated. The overall model was significant (χ^2^ (5) = 13.77; *p* = 0.017), explaining 24% of the variance in BAR+ classification (Nagelkerke R^2^ = 0.24). A direct effect of dissociation on affective vulnerability was observed (b = 1.21; SE = 0.60; *p* = 0.045; 95% CI [0.03–2.39]), corresponding to an odds ratio (OR) = 3.35 (95% CI [1.03–10.91]). Dissociation also had a significant positive effect on attentional impulsivity (b = 3.74; SE = 0.97; *p* < 0.001), with higher mean BIS-11 attentional scores among DES+ participants (21.76 ± 3.62) compared to DES− individuals (17.63 ± 4.07; *p* < 0.001). The model accounted for 28.6% of the variance in attentional impulsivity (R^2^ = 0.286; F (4,65) = 6.50; *p* < 0.001). Attentional impulsivity, however, did not significantly predict BAR+ status (b = 0.073; SE = 0.072; *p* = 0.311). The indirect effect of dissociation on affective vulnerability via attentional impulsivity was also non-significant (β = 0.27; Boot SE = 0.33; 95% Boot CI [−0.31, 1.05]) (Figure 3).

Overall, the model indicated a direct effect of dissociation on affective vulnerability, with attentional impulsivity not serving as a significant mediator.

## 4. Discussion

The analyzed sample reflected the clinical target population of a specialized outpatient service focused on the early assessment of emerging psychopathology in adolescence and young adulthood. The age distribution was weighted toward adolescence (median age = 17; 51.9% < 18 years), consistent with epidemiological evidence indicating that a substantial proportion of psychiatric conditions see onset before 18 years of age [47,48]. The sample showed a slight predominance of female participants, a pattern commonly observed in youth mental health services [49,50]. The sample was socio-culturally homogeneous, and referrals were primarily community-based, which is consistent with the role of the clinic as an intermediary service between primary and specialized care within low-threshold early-intervention pathways [51]. Clinically significant dissociative symptomatology was identified in 42.9% of participants, a prevalence that appears broadly comparable to that reported in other specialized clinical populations [29,52,53,54]. Diagnostic evaluation using the SCID-D identified dissociative disorders in 22.2% of participants, in line with evidence from adolescent and young adult outpatient samples assessed with structured dissociation interviews [55,56]. DES+ participants were predominantly female and presented a lower mean age than DES− participants, although neither difference reached statistical significance [57]. Dissociative symptomatology in youth has been associated with higher overall clinical severity, increased risk of self-harm and suicidality, and poorer psychosocial functioning [58,59,60,61,62], which supports the clinical relevance of systematic screening for dissociation in youth mental health services. The prevalence of BAR status in this sample was 44.7%, which appears higher than rates reported in the original BAR criteria and SIBARS validation studies in help-seeking adolescents and young adults, where approximately 10–13% met bipolar at-risk criteria [63,64]. This difference may reflect the second-level nature of the outpatient service [12], which receives referrals exclusively from other healthcare professionals, including private psychiatrists, community mental health services, child neuropsychiatry services, neurologists, and general practitioners, through whom help-seeking individuals may access care, thereby contributing to a clinically enriched sample with overrepresentation of affective instability, emotional dysregulation, and broader psychopathology dimensions. Furthermore, the use of subthreshold bipolar vulnerability criteria may capture a wider spectrum of early affective dysregulation, particularly in clinical stages characterized by fluctuating mood symptoms and transdiagnostic presentations [65,66]. Elevated BAR prevalence should be interpreted not as an epidemiological estimate but as an indicator of enriched bipolar vulnerability within a clinically referred youth cohort. In our sample, BAR+ status was associated with greater psychopathological burden in help-seeking youths [11,13]. Indeed, lifetime anxiety disorders, lifetime antidepressant treatment, loneliness and NSSI were more frequent among BAR+ participants, mirroring the clinical complexity reported in previous BAR-related cohorts. Bivariate analyses indicated a significant association between dissociative symptomatology and subthreshold affective vulnerability, with 58.8% of DES+ participants meeting BAR+ criteria compared with 28.6% of DES− individuals. Similar associations have been reported, suggesting a clinical overlap between dissociative features and affective instability in mood-disordered and at-risk populations [18,67]. In particular, dissociative features have been reported at higher rates in individuals with BD compared with both healthy controls and individuals with unipolar depression and have been associated with more complex clinical courses and increased suicidality [68]. DES+ individuals were more likely to meet BAR+ criteria and showed higher cyclothymic temperament scores than DES− participants. BAR+ participants reported higher rates of family history of affective disorders and parental psychiatric conditions, consistent with the familial risk component embedded in the BAR Group III classification and with evidence for familial aggregation of bipolar spectrum liability in youth and help-seeking samples [11]. Measures of cyclothymic and irritable temperament were elevated in BAR+ participants and in those with dissociative symptomatology, highlighting the associations between these temperamental dimensions and affective psychopathology [69]. Cyclothymic temperament has been associated with affective lability and mood variability, and with higher risk for bipolar spectrum conditions and related negative outcomes in at-risk and clinical populations [70,71]. Lifetime anxiety disorders were highly prevalent among BAR+ participants, and appeared more frequently than in some previous studies [11,71]. Anxiety during adolescence has been described as a frequent clinical feature in individuals presenting with affective instability and sleep–wake disturbances and has been examined as a potential early indicator of bipolar spectrum vulnerability [72,73]. Although genetic risk indices for anxiety show weaker associations with bipolar disorder compared with those for bipolar disorder or attention-deficit/hyperactivity disorder [74], clinically significant anxiety has been reported to retain prognostic relevance in clinical samples [71]. NSSI was observed more frequently among BAR+ participants, with prevalence comparable to that reported in adolescent bipolar disorder cohorts [6]. In prior studies, NSSI has been associated with depressive symptom severity, mixed features, and suicidal ideation in bipolar populations and is commonly considered an indicator of increased clinical complexity [6,75]. BAR+ participants also reported higher rates of lifetime exposure to antidepressant treatments. Previous studies have reported associations between antidepressant-resistant depressive presentations and latent bipolar features [76,77]. In individuals with bipolar-spectrum vulnerability, antidepressant exposure has been discussed in relation to the occurrence of affective switching and hypomanic symptoms [78]. Trait impulsivity was elevated in the BAR+ group. BAR+ participants scored higher on the BIS-11 total, attentional, and non-planning subscales, consistent with prior reports describing impulsivity as a relatively stable temperamental dimension associated with bipolar spectrum disorders [79,80]. Higher levels of impulsivity were observed in association with earlier age at onset, emotional dysregulation, and NSSI in previous studies. Attentional impulsivity, reflecting difficulties in sustained cognitive focus, and non-planning impulsivity, reflecting reduced future-oriented planning, were both elevated, in line with the temperamental profile described in Group II of the BAR criteria [11]. Within the BAR+ subgroup, clinically significant dissociative symptomatology was associated with more frequent reports of lifetime traumatic events, particularly early-life trauma, greater loneliness, more frequent lifetime antidepressant exposure, higher depressive symptom severity, and higher anxious temperament scores. The 100% rate of lifetime antidepressant exposure observed in the BAR+/DES+ subgroup likely reflects the high prevalence of depressive and/or anxious presentations that had previously managed with antidepressant treatment, without achieving a full clinical response [18]. These findings support the clinical relevance of comprehensive assessment of both bipolar-spectrum vulnerability and dissociative symptomatology in youths presenting with complex affective symptoms. Loneliness was more prevalent among BAR+ participants and were further elevated within the BAR+/DES+ subgroup. Prior studies have reported associations between dissociative symptoms, reduced psychosocial functioning, and lower quality of life, suggesting that dissociation may contribute to interpersonal difficulties, thereby exacerbating perceived loneliness and impairing overall psychosocial adjustment [62]. Dissociation and emotional dysregulation have also been examined in relation to perceived stress and withdrawal tendencies in youth and clinical populations [61]. This pattern suggests that dissociative symptoms may mark a subgroup with greater clinical burden among youths presenting with bipolar at-risk status [12]. Given the small sample size, the binary logistic regression should be considered exploratory and interpreted cautiously as hypothesis-generating; nonetheless, it yielded consistent results, with DES+ participants showing a higher probability of BAR+ classification after adjustment for age, gender, and loneliness. Dissociative symptomatology retained a significant association with bipolar at-risk status, with DES+ participants being approximately three times more likely to meet BAR+ criteria than DES− individuals. Exploratory analyses examined attentional impulsivity as a potential intermediate variable between dissociative symptomatology and bipolar at-risk status [81]. Dissociation retained a direct association with affective vulnerability, indicating a higher likelihood of meeting BAR+ criteria among DES+ participants. Dissociation was also associated with higher attentional impulsivity; however, the indirect pathway from dissociation through attentional impulsivity to BAR+ status did not reach statistical significance. Although attentional impulsivity and dissociation are correlated, attentional impulsivity did not account for the association between dissociative symptomatology and affective vulnerability systems [26,82]. Overall, the BAR+/DES+ subgroup was characterized by the co-occurrence of dissociative symptoms, greater depressive burden, higher anxious temperament and increased loneliness and reported a higher frequency of type I or II traumatic events, largely reflecting adverse childhood experiences, paralleling evidence that bipolar-spectrum psychopathology is marked by pronounced affective reactivity, variability, and instability in daily life [15,83]. These features could delineate a clinically complex profile within the bipolar-risk spectrum, in line with a transdiagnostic approach to the characterization of affective vulnerability in help-seeking youths. The findings suggest that assessment for dissociative symptoms may be a useful component of early evaluation protocols for youths presenting with bipolar-spectrum vulnerability.

### Limitations

This study has several limitations that should be considered when interpreting the findings. The small sample size and the cross-sectional design preclude causal inference regarding the association between clinically significant dissociation (DES+) and subthreshold affective vulnerability (BAR+), as well as any assessment of the predictive value of dissociation for subsequent bipolar spectrum outcomes. Accordingly both the regression and mediation models should be considered as exploratory and hypothesis-generating. The help-seeking sample was recruited from a second-level specialized outpatient service and was intentionally enriched for clinical complexity, which may limit generalizability to community or primary-care populations and reduce statistical power for detecting small effect sizes. Most measures relied on self-report instruments, which may be subject to recall and response biases, especially for the assessment of dissociative experiences, affective symptoms, impulsiveness, and affective temperaments. Missing data across self-report instruments resulted in slight variations in sample size across analyses; available-case approaches were therefore adopted without imputation, which may have influenced statistical estimates. Objective or performance-based assessments of emotional regulation were not included. Finally, the absence of longitudinal follow-up data precludes evaluation of the temporal stability of the observed findings and their association with later diagnostic trajectories or clinical outcomes, thereby limiting interpretation of prognostic relevance.

## 5. Conclusions

This study examined the prevalence of bipolar at-risk status and clinically significant dissociative symptomatology, as well as their association, in a help-seeking sample of adolescents and young adults. The sample was characterized by elevated psychopathological burden, with substantial proportions of participants showing clinically significant dissociative symptomatology and bipolar at-risk status. Within the bipolar at-risk subgroups, the co-occurrence of clinically significant dissociative symptoms identified youths with greater affective burden, loneliness and higher anxious temperament. Overall, the findings indicate that the specialized outpatient setting captures a clinically complex subgroup within the broader population of youth presenting with affective and emotional difficulties, and support the relevance of incorporating dissociation-focused assessment into early evaluation protocols for adolescents and young adults. Such an approach may contribute to improved clinical characterization and risk stratification of help-seeking populations within specialized youth mental health services.

## Figures and Tables

**Table 1:** Comparison of sociodemographic, clinical, and psychopathological characteristics of the BAR+ (n = 34) and BAR− (n = 43) populations in our sample.

Variable (Yes Listed)	BAR− (%)	BAR+ (%)	*p*	OR (95% CI)
Sociodemographic				
Age < 18 years	52.4	52.9	1.000	1.02 (0.41–2.53)
Female gender	50.0	67.6	0.266	-
Italian nationality	100	97.6	1.000	1.83 (1.49–2.85)
Support teacher	11.9	5.9	0.614	0.46 (0.08–2.55)
School refusal	17.1	21.9	0.828	1.46 (0.42–4.37)
Working	7.1	14.7	0.489	2.24 (0.49–10.14)
NEET status	16.7	23.5	0.647	1.54 (0.49–4.78)
Living with family of origin	85.7	76.5	0.462	0.54 (0.17–1.75)
Clinical				
Loneliness	21.4	48.5	*0.026*	3.45 (1.26–9.42)
Social isolation	31.0	52.9	0.088	2.51 (0.98–6.42)
Hyperconnectedness	23.5	16.7	0.933	0.65 (0.12–3.46)
History of trauma (any)	35.7	50.0	0.307	1.80 (0.72–4.53)
Current environmental conflict	45.2	55.9	0.489	1.53 (0.62–3.81)
First contact with psychiatric services	11.9	11.8	1.000	0.99 (0.24–4.00)
Psychiatric familiar history	72.5	78.1	0.784	1.35 (0.46–4.02)
Familiar history of affective disorders	36.8	65.6	*0.031*	3.27 (1.22–8.75)
Parental history of affective disorders	37.5	68.8	*0.016*	3.67 (1.37–9.81)
Lifetime anxiety disorders	54.8	79.4	*0.045*	3.19 (1.14–8.92)
Lifetime eating disorders	28.6	26.5	1.000	0.90 (0.33–2.48)
Lifetime neurodevelopmental disorders	11.9	23.5	0.302	2.28 (0.67–7.75)
Lifetime suicide attempts	7.1	17.6	0.293	2.79 (0.64–12.10)
Lifetime NSSI	31.0	73.5	*<0.001*	6.20 (2.27–16.91)
Lifetime substance abuse	29.3	32.4	0.971	1.16 (0.43–3.09)
Lifetime alcohol abuse	12.2	14.7	1.000	1.24 (0.33–4.71)
Lifetime legal involvement	0.0	2.9	0.447	0.44 (0.34–0.57)
Lifetime antidepressant treatment	9.5	29.4	*0.037*	3.96 (1.11–14.05)
Lifetime mood stabilizer treatment	17.1	17.6	1.000	1.04 (0.31–3.45)
Lifetime antipsychotic treatment	26.2	44.1	0.163	2.22 (0.85–5.84)
Lifetime BDZ treatment	34.1	32.4	1.000	0.92 (0.35–2.42)
	BAR− (mean, SD)	BAR+ (mean, SD)	*p*-value	Cohen’s d
Age at onset	12.55 (3.72)	12.15 (4.91)	0.687	0.09
Age at first psychiatric treatment	15.55 (3.57)	14.82 (4.99)	0.527	0.17
Psychopathological (test scoring)				
BDI-II	18.60 (13.83)	25.38 (14.10)	0.082	0.49
MADRS	11.13 (7.58)	17.60 (8.90)	*0.005*	0.79
KMDRS	10 (5.45)	15.44 (8.45)	0.126	0.03
STAI-Y1	52.60 (14.92)	53.18 (10.65)	0.930	0.02
briefTEMPS cyclothymic	15.87 (5.62)	25.75 (5.72)	*<0.001*	1.73
briefTEMPS irritable	12.80 (4.99)	20.37 (7.21)	*0.001*	1.23
briefTEMPS anxious	17.13 (5.62)	23.11 (5.91)	*0.004*	1.02
BIS-11 total	16.84 (12.36)	71.82 (10.14)	*0.031*	0.52
BIS-11 attentional	18.16 (4.73)	20.67 (3.56)	*0.014*	0.59
BIS-11 motor	20.57 (5.91)	21.85 (5.67)	0.359	0.22
BIS-11 non-planning	27.22 (3.92)	29.70 (5.38)	*0.03* *0*	0.54
	BAR− (median, IQR)	BAR+ (median, IQR)	*p*-value	r
HAM-D	7.00 (8)	11.00 (11)	0.073	0.21
HAM-A	13.50 (13)	17.00 (17)	0.305	0.12
STAI-Y2	54.50 (28)	58.00 (16)	0.973	0.003
briefTEMPS-M depressive	20.00 (10)	27.00 (10)	*0.038*	0.35
briefTEMPS-M hyperthymic	15.00 (9)	17.00 (8)	0.595	0.09
PQ-B symptom total score	4.50 (9)	10.00 (12)	*0.015*	0.37

Notes: BAR = Bipolar-at-risk; BDI-II = Beck Depression Inventory, version 2; BIS-11 = Barratt Impulsiveness Scale, 11 items; briefTEMPS-M: Brief Temperament Evaluation of Memphis, Pisa and San Diego; HAM-A = Hamilton Anxiety Scale; HAM-D = Hamilton Depression Scale; KMDRS = Koukopoulos Mixed Depression Rating Scale; MADRS = Montgomery–Åsberg Depression Rating Scale; NEET = Not in education, employment or training; NSSI = Non-suicidal self-injury; PQ-B = Prodromal Questionnaire–Brief; SD = Standard Deviation; STAI-Y = State–Trait Anxiety Inventory.

**Table 2:** Comparison of the psychopathological characteristics, evaluated with clinical scales, between BAR patients with (BAR+/DES+) and without (BAR+/DES−) clinically significant dissociation.

Variable	BAR+/DES− (Mean, SD)	BAR+/DES+ (Mean, SD)	*p*-Value	Cohen’s d
BDI-II	16.67 (12.33)	30.60 (12.72)	*0.015*	1.09
MADRS	14.43 (6.87)	21.64 (9.84)	*0.042*	0.88
HAM-D	13.13 (5.79)	11.00 (6.16)	0.426	0.35
KMDRS	12.00 (7.78)	18.20 (8.11)	0.120	0.78
STAI-Y1	52.31 (10.74)	53.75 (10.88)	0.711	0.13
BIS-11 attentional	19.15 (3.53)	21.65 (3.30)	*0.047*	0.73
BIS-11 motor	21.15 (5.15)	22.30 (6.07)	0.579	0.20
BIS-11 non-planning	28.23 (6.19)	30.65 (4.69)	0.212	0.45
BIS-11 total	67.77 (10.17)	74.45 (9.45)	0.063	0.69
briefTEMPS-M depressive	23.13 (6.69)	26.20 (4.92)	0.277	0.53
briefTEMPS-M cyclothymic	23.00 (6.39)	27.58 (4.62)	0.078	0.86
briefTEMPS-M irritable	19.56 (6.80)	21.10 (7.84)	0.654	0.21
briefTEMPS-M anxious	19.44 (5.34)	26.40 (4.35)	*0.006*	1.43
	BAR+/DES− (median, IQR)	BAR+/DES+ (median, IQR)	*p*-value	r
HAM-A	16 (24)	17 (20)	0.424	0.14
STAI-Y2	57 (42)	58.5 (16)	0.083	0.30
briefTEMPS-M hyperthymic	18.5 (11)	13 (8)	0.884	0.04
PQ-B symptom total score	11 (15)	9.5 (11)	0.631	0.10

Notes: BDI-II = Beck Depression Inventory, version 2; BAR = Bipolar-at-risk; BIS-11 = Barratt Impulsiveness Scale, 11 items; briefTEMPS-M: Brief Temperament Evaluation of Memphis, Pisa and San Diego; DES = Dissociative Experience Scale; HAM-A = Hamilton Anxiety Scale; HAM-D = Hamilton Depression Scale; KMDRS = Koukopoulos Mixed Depression Rating Scale; MADRS = Montgomery–Åsberg Depression Rating Scale; PQ-B = Prodromal Questionnaire–Brief; SD = Standard Deviation; STAI-Y = State–Trait Anxiety Inventory.

**Table 3:** Logistic regression considering Bipolar at-risk (BAR) status as the dependent variable and gender, age, loneliness, and dissociation as covariates.

Variables in Equation	Wald	*p*-Value	OR (95% CI)
Female gender	2.353	0.125	2.293 (1.081–6.739)
Age	0.567	0.452	1.065 (0.794–6.624)
Loneliness	4.919	*0.027*	3.397 (0.153–10.012)
Dissociation	4.677	*0.031*	3.165 (1.114–8.991)

Chi-square: 14.023; df: 4; *p* = 0.007. Notes: CI = confidence interval; OR = odds ratio.

## Data Availability

The data presented in this study are available on request from the corresponding author due to ethical reasons.
